# The Infratrochlear Nerve Block Reduces the Incidence of Postoperative Nausea Vomiting in Pediatric Patients Undergoing Strabismus Surgery—A Retrospective Study

**DOI:** 10.3390/biomedicines13030580

**Published:** 2025-02-25

**Authors:** Chung-Sik Oh, Hyun Jin Shin, Seon-Ju Park, Seong-Hyop Kim, Yea-Ji Lee

**Affiliations:** 1 Department of Anesthesiology and Pain Medicine, Konkuk University Medical Center, Seoul 05030, Republic of Korea; ohcsik@naver.com (C.-S.O.); tjswn9410@naver.com (S.-J.P.); yshkim75@daum.net (S.-H.K.); 2 Research Institute of Medical Science, Konkuk University School of Medicine, Seoul 05030, Republic of Korea; shineye@kuh.ac.kr; 3 Institution of Biomedical Sciences and Technology, Konkuk University School of Medicine, Seoul 05030, Republic of Korea; 4 Department of Ophthalmology, Konkuk University Medical Center, Seoul 05030, Republic of Korea; 5 Department of Medical Education, Konkuk University School of Medicine, Seoul 05030, Republic of Korea; 6 Department of Infection and Immunology, Konkuk University School of Medicine, Seoul 05030, Republic of Korea

**Keywords:** postoperative nausea and vomiting, ophthalmologic surgical procedures, nerve block, reflex oculocardiac, pain, postoperative

## Abstract

**Background/Objectives**: Strabismus surgery in pediatric patients is associated with a high incidence of postoperative nausea and vomiting (PONV). Patients showing pain are more prone to develop PONV. As the infratrochlear nerve (ITN) block can ameliorate perioperative pain following strabismus surgery, we hypothesized that ITN block may influence PONV in pediatric patients undergoing strabismus surgery. **Methods**: The medical charts of pediatric patients older than 2 years with exotropia who underwent strabismus surgery under general anesthesia, with or without ITN block, were reviewed retrospectively. The incidence of PONV, intraoperative surgical pleth index (SPI), state entropy (SE), response entropy (RE), the changes in hemodynamics, and perioperative use of metoclopramide and ketoprofen were investigated. **Results**: The study population comprised 116 patients (58 for the No-block group vs. 58 for the ITN group). The incidence of PONV was significantly lower in the ITN block group compared to the No-block group (5.2% vs. 22.4%, respectively; *p* = 0.015). The SPI at conjunctiva incision, muscle dissection and traction were significantly lower in the ITN block group than in the No-block group. SE was comparable between the two groups, but RE at muscle dissection and traction was significantly lower in the ITN block group than in the No-block group. The use of metoclopramide and ketoprofen was also lower in the ITN block group than in the No-block group. **Conclusions**: ITN block reduced PONV as well as perioperative pain in pediatric patients undergoing strabismus surgery.

## 1. Introduction

The oculocardiac reflex (OCR) during strabismus surgery is a type of vagal reflex that results in intraoperative bradycardia and related complications [1,2]. The severity of bradycardia due to OCR exhibits interindividual variation, and bradycardia is dangerous in pediatric patients because the cardiac output is more reliant on heart rate in children than in adults [3]. Therefore, reducing OCR during strabismus surgery is important in pediatric patients. A number of methodologies have been developed to ameliorate OCR during strabismus surgery, including premedication with anticholinergic agents, optimal sedation, local hypothermia, and reduced tension on ocular muscle during surgery [4,5]. In addition to these methods, an infratrochlear nerve (ITN) block was reported to reduce intraoperative OCR during strabismus surgery [6].

Postoperative nausea and vomiting (PONV) is a major postoperative complication that can be influenced by various factors, including the type of surgery and anesthesia used. [7]. Previous studies have demonstrated that patients who experience OCR during strabismus surgery are more likely to develop PONV [8,9,10]. These results highlight the necessity of mitigating OCR during strabismus surgery to minimize the risk of PONV. However, there have been few studies that evaluated the impact of ITN block on PONV after strabismus surgery. Therefore, we hypothesized that ITN block would reduce PONV after strabismus surgery, and the present study was designed to evaluate the effects of ITN block on PONV in pediatric patients undergoing strabismus surgery.

## 2. Materials and Methods

### 2.1. Study Population

The protocol of this retrospective study received Institutional Review Board approval (approval number KUH2024-11-016, granted by the Institutional Review Board of Konkuk University Medical Center, Seoul, Korea; Chairperson Prof SH. Lee). The retrospective review of the medical charts of pediatric patients over 2 years old with exotropia who underwent strabismus surgery involving unilateral medial rectus resection (MR) and lateral rectus recession (LR) under general anesthesia at Konkuk University Medical Center was conducted. Patients categorized as American Society of Anesthesiologists (ASA) class I or II (Table 1) [11] were included in the present study. Patients with a history of previous PONV, concurrent other ocular disease, congenital cardiac disease or central nervous disease, or previous strabismus surgery were excluded.

### 2.2. Anesthesia Technique

The anesthesia techniques were standardized according to the institutional protocol, as described below. No patient received preanesthetic medication. Anesthesia was induced after establishing routine non-invasive monitoring, including electrocardiography, non-invasive arterial blood pressure, pulse oximetry, temperature, oxygen saturation, surgical pleth index (SPI), state entropy (SE), and response entropy (RE). The mean blood pressure (MBP) and heart rate (HR) were recorded continuously during anesthesia. Preoxygenation was applied in all patients with 100% oxygen. Intravenous thiopental sodium 5 mg/kg was administered intravenously to induce anesthesia. After loss of consciousness, mask ventilation was confirmed, and intravenous rocuronium 0.6 mg/kg was administered. Tracheal intubation was performed, and pressure-controlled ventilation was applied to achieve a tidal volume of 6 mL/kg. The respiration rate was adjusted to achieve end-tidal carbon dioxide levels of 35 to 40 mmHg. Anesthesia was maintained with inhaled sevoflurane in 40% oxygen by titrating the RE and SE from 40 to 60 in all patients during surgery. At the end of the surgery, inhaled sevoflurane was stopped, and residual neuromuscular blockade was antagonized with intravenous neostigmine 0.03 mg/kg and glycopyrrolate 0.008 mg/kg. After tracheal extubation, the patient was transferred to the postanesthesia care unit.

### 2.3. ITN Block and Strabismus Surgery

After the induction of anesthesia, strabismus surgery was performed without regional block in patients in the No-block group and with regional block in the ITN block group. The ITN block was performed by transcutaneous injection of 1 mL of 2% lidocaine with epinephrine, which was diluted to 1:100,000, using a 30-gauge needle at the medial orbital rim, just above the lacrimal caruncle. After the block, unilateral MR and LR were performed in all patients’ non-dominant eyes. Strabismus surgery was performed in the following order: stage I, a conjunctival incision was made through the inferior nasal fornix, and an incision was made in Tenon’s capsule and the intermuscular septum; stage II, hooking and dissection of the MR, as well as exposure and dissection of the check ligament and the intermuscular septum from the muscle capsule; stage III, application of traction to the MR for 1 min, with stretching of 10 mm in all cases to ensure that the stimulus applied to the muscle was as uniform as possible.

### 2.4. Management of PONV and Postoperative Pain

According to our institutional protocol, postoperative PONV and pain were assessed in all pediatric patients after strabismus surgery by the responsible medical staff. If the patients showed symptoms of PONV, such as nausea or vomiting episodes, intravenous metoclopramide 0.15 mg/kg was administered, and the patient was hydrated with intravenous fluid at 10 mL/kg/h. Patients with postoperative pain, classified as a Wong-Baker Faces (WBF) scale score ≥ 5 [12], received intramuscular ketoprofen 1.0 mg/kg.

### 2.5. Management of OCR

OCR was defined as a sudden decrease in HR of >15% from baseline during the entire surgical procedure. In cases with a sudden decrease of >30% in HR from baseline, the surgical procedure was stopped to ameliorate the bradycardia. Surgical procedures were resumed when the HR recovered. Atropine 0.01 mg/kg was administered intravenously if HR decreased to <40 beats/min or HR did not recover within 5 s after OCR.

### 2.6. Clinical Measurement

The overall incidence of PONV was subdivided into nausea and vomiting episodes, and the incidence of metoclopramide use to reduce PONV was obtained from medical records. The overall incidence of intraoperative OCR and the incidence of intraoperative atropine use were obtained from medical records. All data were acquired at a total of five time points defined as follows: baseline was defined as the time of hemodynamic stability after induction of anesthesia and surgical preparation; stages I to III were defined as each stage of MR; and muscle suture was defined as the point at which the MR was finalized. The values of SPI, HR, and MBP at each time point were obtained from the medical records. The incidence of ketoprofen use for postoperative pain control was also obtained from medical records. In addition, the end-tidal concentration of sevoflurane and intraoperative entropy values, including RE and SE at each time point, were obtained from medical records.

### 2.7. Statistical Analysis

The primary endpoint of the present study was the cumulative incidence of PONV within 24 h after strabismus surgery, and the secondary endpoint was the intergroup difference in the SPI value during strabismus surgery. Based on the previous literature [6,13,14], we assumed that the incidence of PONV after strabismus surgery would be 50% and that ITN block would reduce PONV by 50% after strabismus surgery. The calculated sample size for the primary endpoint was 58 in each group to obtain a power of 0.8 and an α value of 0.05. Therefore, we collected data from 116 patients who underwent strabismus surgery.

For continuous variables, the distribution of the data was first evaluated for normality using the Shapiro-Wilk test. An independent two-tailed *t*-test was used to compare the means of continuous variables with normal distributions. The Mann-Whitney U test was used for the analysis of data without a normal distribution. The chi-square test was used to compare categorical variables between the two groups. Intragroup hemodynamic changes and intergroup differences over time were analyzed by repeated-measures variance analysis. If a significant difference was noted, the independent two-tailed *t*-test or Mann–Whitney U test was used to compare differences between groups with Bonferroni correction. Normally distributed continuous data are presented as the mean ± standard deviation. Continuous data, without a normal distribution, are presented as the median (interquartile range, IQR). For categorical variables, the number of patients (*n*) and proportion (%) were calculated. All calculations were performed using R version 4.4.1 (R Foundation for Statistical Computing, Vienna, Austria). In all analyses, *p* < 0.05 was taken to indicate statistical significance.

## 3. Results

**Figure 1:**
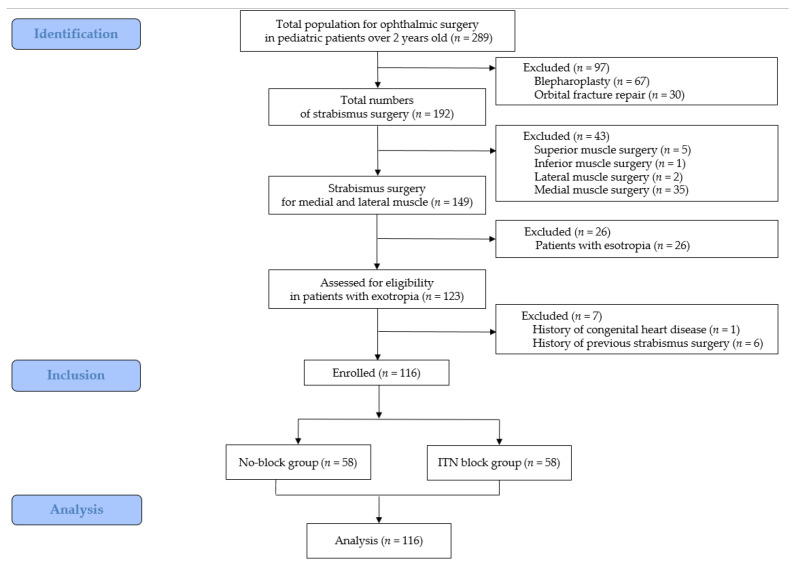
Study flow diagram.

Data from a total of 123 consecutive patients who underwent strabismus surgery for exotropia between July 2016 and December 2017 were screened for inclusion in the present study. Seven patients were excluded due to a history of congenital cardiac disease (*n* = 1) or a history of previous strabismus surgery (*n* = 6). Therefore, 116 patients were included in the final analysis (58 for the No-block group vs. 58 for the ITN block group) (Figure 1).

The distribution of demographic variables was similar between the two groups (Table 2). Age, body mass index, anesthesia and operation duration were comparable between the two groups (Table 2).

The overall incidence of PONV after strabismus surgery was 13.8% in the total study population (16 of 116 patients) and was significantly lower in the ITN block group than the No-block group (5.2% vs. 22.4%, respectively; *p* = 0.015) (Table 3). The incidence of nausea episodes was significantly lower in the ITN block group than in the No-block group (3.4% vs. 19.0%, respectively; *p* = 0.019). The incidence of vomiting episodes was lower in the ITN block group than in the No-block group, but the difference was not statistically significant (5.2% vs. 15.5%, respectively; *p* = 0.127). The incidence of postoperative metoclopramide use was significantly lower in the ITN block group than in the No-block group (5.2% vs. 22.4%, respectively; *p* = 0.015) (Table 3). The overall incidence of OCR during strabismus surgery was 30.2% in the total study population (35 of 116 patients) and was significantly lower in the ITN block group than in the No-block group (17.2% vs. 43.1%, respectively; *p* = 0.005). The incidence of atropine use during strabismus surgery was lower in the ITN block group than in the No-block group, but the difference was not statistically significant (3.4% vs. 13.8%, respectively; *p* = 0.098) (Table 3).

The SPI values at baseline and at muscle suture were comparable between the two groups (Table 4). However, the SPI values at stages I, II and III of MR were significantly lower in the ITN block group than in the No-block group (34.9 ± 8.5 vs. 40.1 ± 7.9, respectively, at stage I, *p* = 0.001; 42.7 ± 9.9 vs. 42.7 ± 9.9, respectively, at stage II, *p* = 0.002; 38.0 [35.0 to 44.0] vs. 42.0 [35.0 to 48.0], respectively, at stage III, *p* = 0.014) (Table 4). Postoperative ketoprofen use was significantly lower in the ITN block group than in the No-block group (6.9% vs. 24.1%, respectively; *p* = 0.021) (Table 4).

**Figure 2:**
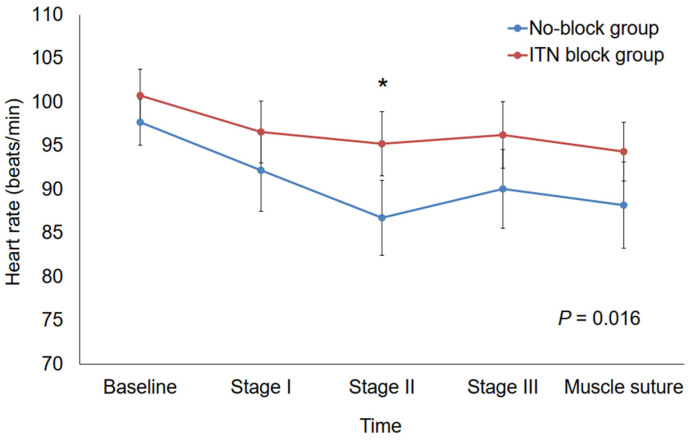
Intraoperative changes in heart rate. (* *p* = 0.008). ITN, infratrochlear nerve; Baseline, the time of hemodynamic stability after anesthesia induction and surgical preparation; stage I, conjunctiva incision during medial rectus resection; stage II, muscle dissection during medial rectus resection; stage III, muscle traction during medial rectus resection; muscle suture, the point at which medial rectus resection was finalized.

**Figure 3:**
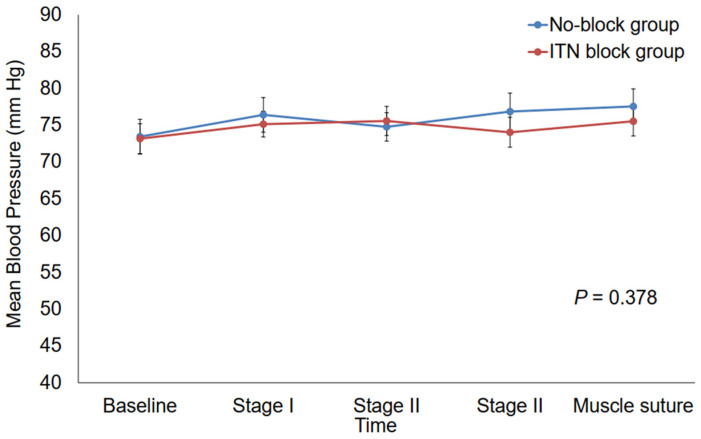
Intraoperative changes in mean blood pressure. ITN, infratrochlear nerve; Baseline, the time of hemodynamic stability after anesthesia induction and surgical preparation; stage I, conjunctiva incision during medial rectus resection; stage II, muscle dissection during medial rectus resection; stage III, muscle traction during medial rectus resection; muscle suture, the point at which medial rectus resection was finalized.

The changes in HR during strabismus surgery were significantly greater in the ITN block group than in the No-block (*p* = 0.016) (Figure 2). In particular, HR at stage II of MR was significantly higher in the ITN block group than in the No-block group (*p* = 0.008) (Figure 2). On the other hand, MBP during strabismus surgery was not significantly different between the two groups (*p* = 0.378) (Figure 3).

The end-tidal sevoflurane concentration at each stage of MR showed no significant intergroup differences (Table 5). The value of SE at each stage of MR and the values of RE at baseline, stage I, and muscle suture were also comparable between the two groups. However, the values of RE values at stage II and III of MR were significantly higher in the No-block group than in the ITN group (55.5 [50.0 to 58.0] vs. 50.0 [47.0 to 57.0], respectively, at stage II, *p* = 0.027; 54.0 [49.0 to 59.0] vs. 51.0 [45.0 to 58.0], respectively, at stage III, *p* = 0.040; respectively) (Table 5).

## 4. Discussion

This study showed that the ITN block reduced the incidence of PONV and the requirement for postoperative metoclopramide use in pediatric patients undergoing strabismus surgery. It also reduced the overall incidence of intraoperative OCR, the intraoperative SPI value from stage I to III of MR changes in HR during strabismus surgery, and postoperative ketoprofen use in pediatric patients undergoing strabismus surgery. The values of RE at stages II and III were lower after ITN block despite the concentration of inhaled anesthetics being comparable to that in the No-block group during strabismus surgery.

The known risk factors for PONV are female, young age, volatile anesthetics, opioids, long duration of anesthesia, and some types of surgery, including gynecological, laparoscopic, and strabismus surgery [15,16,17]. There are several factors associated with PONV in the present study, such as young age, volatile anesthetics, and strabismus surgery. We used sevoflurane as an anesthetic because volatile anesthetics are traditionally preferred in pediatric patients due to their easy and rapid reversibility [18]. However, volatile anesthetics may induce more PONV rather than intravenous anesthetics [16], and high concentrations of volatile anesthetics increase the incidence of PONV compared with low concentrations of volatile anesthetics [19]. In addition, a previous study showed that the incidence of PONV was higher in pediatric patients than in adult patients [20]. Finally, strabismus surgery was also considered to be the highest risk in terms of PONV among surgical procedures in pediatric patients [13,21]. Although we did not use opioids, one of the major risk factors for PONV, for anesthesia and pain control, the above three factors may have contributed to PONV in the present study. As PONV is associated with postoperative complications, prolonged hospital stay, unexpected hospital admission, and increased medical costs [22,23,24,25], it is crucial to reduce PONV in pediatric patients undergoing strabismus surgery by using volatile anesthetics. Numerous methods have been recommended for prophylaxis and therapeutic management of PONV, including 5-hydroxytryptamine (HT)_3_ receptor antagonists, corticosteroids, antihistamines, and antidopaminergic agents [7,26,27]. However, the medications used for PONV prophylaxis should balance the risks and benefits because they are associated with adverse effects such as arrhythmias, including QT-prolongation, reduction of the effectiveness of postoperative analgesics, tumor-lysis syndrome, tissue damage, and extrapyramidal neurological complications [28,29,30,31,32,33]. Therefore, nonpharmacologic methods of PONV prophylaxis can be beneficial in terms of reducing the incidences of adverse effects. Analgesia by regional block has been recommended for PONV prophylaxis because it can reduce intra- and postoperative opioid consumption [34]. From this perspective, a regional block of the ITN would be a good option to ameliorate PONV in pediatric patients undergoing strabismus surgery. Nevertheless, there are few studies on the antiemetic efficacy of ITN block during strabismus surgery. Kim et al. reported that ITN block reduced postoperative pain and emergence agitation but did not reduce PONV in septorhinoplasty [35]. We assume that ITN block may be less effective in preventing PONV in septorhinoplasty because the major factor contributing to PONV in septorhinoplasty is intraoperative bleeding, which leads to aspiration of blood into the gastric space [36]. On the other hand, ITN block may have beneficial effects in reducing PONV after strabismus surgery because the major factor contributing to PONV in strabismus surgery is the enhancement of the trigeminovagal reflex, which affects the vomiting center in the brain [9,10]. Although the ITN block is not directly related to the vagus nerve, it may have indirect effects on PONV by ameliorating nociceptive stimulation from surgical procedures and postoperative pain [35]. Song et al. reported that a high level of nociceptive stimulation from surgical procedures is an independent predictor of PONV [37]. Therefore, amelioration of perioperative nociceptive stimulation can, in turn, ameliorate PONV after surgery. Three findings in the present study suggested that ITN blockade reduced surgical nociceptive stimulation from surgical procedures and postoperative pain. First, the intraoperative SPI was lower after the ITN block during strabismus surgery. The SPI is determined from the photoplethysmographic signals of finger arterioles to detect the balance between nociceptor activation and analgesia [38]. Compared with hemodynamic parameters, the SPI can be used to determine the degree of nociceptive stimulation during surgery under general anesthesia with high accuracy, and a high SPI indicates high nociceptive stimulation during surgery [39]. Therefore, the lower intraoperative SPI observed in our ITN block group indicated amelioration of intraoperative pain. Second, the incidence of postoperative ketoprofen use was low after strabismus surgery with ITN block compared with the No-block group, indicating that ITN block ameliorated postoperative pain. Third, RE during muscle dissection and traction were lower after ITN block, although the concentration of inhaled anesthetic was comparable to that in the No-block group. Entropy monitors the state of the brain based on electroencephalograph (EEG) and frontal electromyography (FEMG) signals, and the SE and RE represent processed EEG and FEMG variables [40]. SE is a parameter used to assess the hypnotic effect of anesthetics on the brain by monitoring EEG, and RE is used to assess the hypnotic effect, as well as to detect the activation of facial muscles by monitoring EEG with FEMG. RE increases as nociceptive stimulation becomes more intense due to increased FEMG activation [41]. The high RE in our No-block group during muscle dissection and traction, indicating relatively strong nociceptive stimulation during MR, indicated that ITN block ameliorated intraoperative pain. We assumed that the above three factors contributed to the reduction of perioperative pain and associated PONV in pediatric patients undergoing strabismus surgery. Several types of conventional regional blocks are used for ophthalmic surgery in adult patients, including peribulbar block, retrobulbar block, and sub-Tenon block [42]. Among these, sub-Tenon block has recently attracted attention due to its improved safety profile compared to other forms of regional block. Ramachandran et al. reported that adjuvant sub-Tenon block under general anesthesia reduced intraoperative OCR and PONV in pediatric patients undergoing strabismus surgery [43]. Nevertheless, the application of these conventional regional blocks in pediatric patients undergoing strabismus surgery has not been recommended because of the potential risk of motor block and other serious complications, including hemorrhage and myotoxicity [44,45]. ITN block, which is a type of superficial block, is associated with a potential risk of eye movement during ocular surgery because it cannot generate a motor block and can only block sensory nerve activity [44,46]. Therefore, the ITN block has been mainly applied in oculoplastic surgery or septorhinoplasty as an adjunct to general anesthesia. However, the fact that ITN block only causes sensory block is actually an advantage in pediatric patients undergoing strabismus surgery under general anesthesia because there is no risk of inadvertent motor block [42]. In addition, there is no risk of bleeding associated with ITN block because only superficial tissues are involved. Considering the low incidence of PONV after ITN block, ITN block may be an option to reduce perioperative pain and PONV in pediatric patients undergoing strabismus surgery, with less risk of complications. However, the ITN block is not a popular block and has limited use in ocular surgery [42], and there is insufficient evidence to support the effect of ITN block in preventing PONV. Therefore, further studies are needed to clarify the effect of ITN block on postoperative pain and PONV.

The present study had several limitations. First, the retrospective design of the present study has potential limitations. However, the included clinical data were well documented in the medical records because the protocol was based on our previous institutional prospective study [6]. The discrepancy in the incidence of OCR between our institutional prospective study and the present retrospective study may be attributed to the different primary endpoints and study periods of the two studies. Arnold et al. revealed a wide range in the incidence of OCR during strabismus surgery, ranging from 10% to 90%, due to the various cut-off values of HR. Consequently, further studies are necessary to evaluate the impact of ITN block on OCR. Second, the values of SPI, SE, RE, and hemodynamic changes during LR were not obtained due to the lack of recording at each time point of LR in the present study. However, previous findings demonstrated that most of the OCR was only seen when the first operated muscle was manipulated because of a counterregulatory effect that outlasted the period of mechanical stimulation of the ocular muscles [47]. Vagal escape due to cholinergic neurotransmitter exhaustion during the first operative muscle manipulation resulted in a reduced vagal response during later operative muscle manipulations [48]. Therefore, we assumed that the incidence of OCR changes in SPI, entropy, and hemodynamics during LR would have been minimal in the present study. Third, the reliability of the SPI in volatile anesthetic-based anesthesia requires further investigation because most studies evaluating the feasibility of the SPI during general anesthesia were performed in cases with total intravenous anesthesia [39]. Similarly, inhalational anesthesia, which was used in the present study, is known to contribute to PONV [7]. Therefore, we cannot extrapolate our result to estimate the incidence of PONV in patients undergoing strabismus surgery with intravenous anesthesia. However, a previous report showed that total intravenous anesthesia with propofol and remifentanil increased the incidence of OCR in pediatric patients undergoing strabismus surgery [49]. The use of opioids such as remifentanil for total intravenous anesthesia may exacerbate opioid-induced bradycardia. In addition, the use of opioids has been shown to contribute to PONV [50]. Therefore, we cannot confirm the benefit of different types of anesthesia in reducing PONV in patients undergoing strabismus surgery.

## 5. Conclusions

ITN block reduced PONV as well as intra- and postoperative pain after strabismus surgery in pediatric patients. Considering the occurrence of complications related to anti-PONV medications and conventional regional block, the ITN block will be a good alternative option to ameliorate PONV in pediatric patients undergoing strabismus surgery.

## Figures and Tables

**Table 1:** ASA physical status classification.

Classification	Definition
ASA I	A normal, healthy patient
ASA II	A patient with mild systemic disease
ASA III	A patient with severe systemic disease
ASA IV	A patient with severe systemic disease that is a constant threat to life
ASA V	A moribund patient who is not expected to survive without the operation
ASA VI	A declared brain-dead patient whose organs are being removed for donor purposes

ASA, American Society of Anesthesiologists.

**Table 2:** Patient demographic data and perioperative clinical characteristics.

	No-Block Group (*n* = 58)	ITN Block Group (*n* = 58)	*p*-Value
Male, *n* (%)	24 (41.4%)	23 (39.7%)	1.000
Age (years)	8.0 (5.0–9.0)	8.0 (5.0–9.0)	0.200
Body mass index (kg/m^2^)	19.0 (17.4–21.8)	18.4 (16.3–20.9)	0.262
Duration of anesthesia (min)	82.5 (75.0–95.0)	85.0 (70.0–105.0)	0.748
Duration of operation (min)	59.6 ± 19.5	61.1 ± 20.8	0.676

Data are expressed as numbers (%), median (IQR), and mean ± standard deviation. ITN, infratrochlear nerve.

**Table 3:** Incidents of PONV and intraoperative OCR.

	No-Block Group (*n* = 58)	ITN Block Group (*n* = 58)	*p*-Value
PONV	13 (22.4%)	3 (5.2%)	0.015
Nausea	11 (19.0%)	2 (3.4%)	0.019
Vomiting	9 (15.5%)	3 (5.2%)	0.127
Metoclopramide use	13 (22.4%)	3 (5.2%)	0.015
OCR	25 (43.1%)	10 (17.2%)	0.005
Atropine use	8 (13.8%)	2 (3.4%)	0.098

Data are expressed as numbers (%). ITN, infratrochlear nerve; OCR, oculocardiac reflex; PONV, postoperative nausea and vomiting.

**Table 4:** Intraoperative SPI and incidence of postoperative ketoprofen use.

	No-Block Group (*n* = 58)	ITN Block Group (*n* = 58)	*p*-Value
SPI			
Baseline	34.0 (28.0–42.0)	36.0 (33.0–38.0)	0.408
Stage I	40.1 ± 7.9	34.9 ± 8.5	0.001
Stage II	42.7 ± 9.9	37.2 ± 8.9	0.002
Stage III	42.0 (35.0–48.0)	38.0 (35.0–44.0)	0.014
Muscle suture	35.5 (26.0–42.0)	36.0 (33.0–42.0)	0.293
WBF scale score	4 (2–4)	2 (2–4)	<0.001
Ketoprofen use	14 (24.1%)	4 (6.9%)	0.021

Data are expressed as median (IQR), mean ± standard deviation, and numbers (%). ITN, infratrochlear nerve; SPI, surgical pleth index; Baseline, the time of hemodynamic stability after anesthesia induction and surgical preparation; stage I, conjunctiva incision during medial rectus resection; stage II, muscle dissection during medial rectus resection; stage III, muscle traction during medial rectus resection; muscle suture, the point at which medial rectus resection was finalized; WBF, Wong-Baker Faces.

**Table 5:** Changes in intraoperative inhaled anesthetic concentration and entropy.

	No-Block Group (*n* = 58)	ITN Block Group (*n* = 58)	*p*-Value
Etc-Sevo (Vol%)			
Baseline	2.0 (1.7–2.3)	2.0 (1.9–2.5)	0.673
Stage I	2.0 (1.9–2.1)	2.0 (1.6–2.4)	0.588
Stage II	2.0 (1.7–2.1)	2.0 (1.9–2.4)	0.108
Stage III	2.0 (1.9–2.2)	2.0 (1.8–2.4)	0.493
Muscle suture	2.0 (1.9–2.3)	2.0 (1.9–2.4)	0.524
SE			
Baseline	52.0 (45.0–55.0)	50.0 (45.0–55.0)	0.888
Stage I	48.0 (44.0–54.0)	48.5 (43.0–53.0)	0.848
Stage II	50.0 (45.0–54.0)	47.0 (45.0–55.0)	0.585
Stage III	50.0 (45.0–55.0)	47.5 (42.0–55.0)	0.215
Muscle suture	51.0 (46.0–55.0)	51.0 (44.0–55.0)	0.666
RE			
Baseline	55.0 (48.0–59.0)	53.0 (48.0–59.0)	0.737
Stage I	53.0 (49.0–59.0)	53.0 (48.0–58.0)	0.386
Stage II	55.5 (50.0–58.0)	50.0 (47.0–57.0)	0.027
Stage III	54.0 (49.0–59.0)	51.0 (45.0–58.0)	0.040
Muscle suture	55.0 (49.0–58.0)	55.0 (48.0–59.0)	0.714

Data are expressed as median (IQR). ITN, infratrochlear nerve; Etc-Sevo, end-tidal concentration of sevoflurane; SE, state entropy; RE, response entropy. Baseline, the time of hemodynamic stability after anesthesia induction and surgical preparation; stage I, conjunctiva incision during medial rectus resection; stage II, muscle dissection during medial rectus resection; stage III, muscle traction during medial rectus resection; muscle suture, the point at which medial rectus resection was finalized.

## Data Availability

The data presented in this study are available at the request of the corresponding author due to restrictions imposed by the Institutional Review Board, which approved the study protocol.
